# Dexmedetomidine Mitigates Microglial Activation Associated with Postoperative Cognitive Dysfunction by Modulating the MicroRNA-103a-3p/VAMP1 Axis

**DOI:** 10.1155/2022/1353778

**Published:** 2022-04-19

**Authors:** Zhichao Wu, Han Wang, Zuan Shi, Yalan Li

**Affiliations:** ^1^Department of Anesthesiology, The First Affiliated Hospital of Jinan University, Guangzhou, 510630 Guangdong, China; ^2^Department of Anesthesiology, Nanchong Central Hospital, The Second Clinical Medical College, North Sichuan Medical College, Nanchong, 637000 Sichuan, China

## Abstract

Surgery-induced microglial activation is critical in mediating postoperative cognitive dysfunction (POCD) in elderly patients, where the important protective effect of dexmedetomidine has been indicated. However, the mechanisms of action of dexmedetomidine during the neuroinflammatory response that underlies POCD remain largely unknown. We found that lipopolysaccharide (LPS) induced substantial inflammatory responses in primary and BV2 microglial cells. The screening of differentially expressed miRNAs revealed that miR-103a-3p was downregulated in these cell culture models. Overexpression of miR-103a-3p mimics and inhibitors suppressed and enhanced the release of inflammatory factors, respectively. VAMP1 expression was upregulated in LPS-treated primary and BV-2 microglial cells, and it was validated as a downstream target of miR-103-3p. VAMP1-knockdown significantly inhibited the LPS-induced inflammatory response. Dexmedetomidine treatment markedly inhibited LPS-induced inflammation and the expression of VAMP1, and miR-103a-3p expression reversed this inhibition. Moreover, dexmedetomidine mitigated microglial activation and the associated inflammatory response in a rat model of surgical trauma that mimicked POCD. In this model, dexmedetomidine reversed miR-103a-3p and VAMP1 expression; this effect was abolished by miR-103a-3p overexpression. Taken together, the data show that miR-103a-3p/VAMP1 is critical for surgery-induced microglial activation of POCD.

## 1. Introduction

Postoperative cognitive dysfunction (POCD), a common postoperative complication in the elderly, is characterized by a progressive deterioration in cognitive function; POCD accelerates development of dementia or neurodegenerative disorders such as Alzheimer's disease (AD) [1–3]. A significant risk factor for POCD is surgical trauma [4], which, in the central nervous system (CNS), induces an exaggerated inflammatory response critical for the manifestation of POCD [5, 6]. In postoperative animals, elevated levels of inflammatory factors in the hippocampus correlate with reduced learning and memory [7]. The cytokines interleukin-1 beta (IL-1*β*) and tumor necrosis factor-alpha (TNF-*α*) are significantly increased in the hippocampus after lipopolysaccharide (LPS) treatment [8, 9]. Surgical trauma induces microglial activation in the brain and increases inflammatory cytokine levels [10, 11]. However, the precise signaling mechanism underlying surgery-evoked modulation of microglial activation remains to be elucidated. Exploring the mechanism of neuroinflammation, including microglial activation and the concomitant inflammatory response, is critical for improving the prognosis of POCD and the identification of therapeutic targets to treat this neurological complication.

MicroRNAs (miRNAs) are widely expressed in the brain and bind to the 3′-untranslated regions (UTR) of target mRNAs to regulate the transcription and translation of target genes [12, 13]. A number of abnormally expressed miRNAs have been detected in Huntington's disease and AD [14, 15]. Specifically, miR-103a-3p is involved in the progression of many types of cancers, including gliomas [16], gastric [17, 18], and colorectal cancers [19]. Particularly, miR-103a-3p plays an important role in the CNS system and neuronal dysfunctions by regulating the downstream targets. miR-103a-3p regulates mitophagy by modulating Parkin in Parkinson's disease [20], oxidative stress, apoptosis, and immune disorder via HMGB1 in oxygen-glucose deprived BV2 cells and after ischemia-reperfusion injury [21]. Hence, miR-103a-3p is related to microglial response; however, the functional significance of miR-103a-3p in microglial activation during POCD is unknown.

Dexmedetomidine, a highly selective *α*2-adrenergic receptor agonist, protects the CNS, heart, liver, and other organs by attenuating the inflammatory response [22]. Dexmedetomidine is neuroprotective in animal models of spinal cord and ischemic brain injury [23] and ameliorates LPS-induced neuronal dysfunction by modulating the phosphoinositide 3-kinase/protein kinase B (PI3K/Akt)/glycogen synthase kinase-3*β* (GSK-3*β*)/collapsin response mediator protein 2 (CRMP-2) [24] pathways. Furthermore, dexmedetomidine suppresses LPS-induced microglial activation and inflammation [25]. The mechanism by which dexmedetomidine modulates microglial activation during POCD, however, is unknown.

In the present study, to mimic POCD, we replicated the cell culture model of LPS-induced microglial activation and established an *in vivo* rat model of surgical trauma. The data show that miR-103a-3p was downregulated during microglial activation and that miR-103a-3p targeted VAMP1 to induce the protective effect of dexmedetomidine on microglial activation in POCD.

## 2. Materials and Methods

### 2.1. Cell Culture and Transfection

Primary microglial cells were cultured from the forebrains of 1-day-old Sprague-Dawley rat pups as previously described [26]. Ten days later, the mixed culture containing microglia, astrocytes, and oligodendrocytes was centrifuged at 220 rpm for 2 h, and supernatants applied to new cell culture plates. Non-adherent cells, primarily astrocytes and oligodendrocytes, were removed after 15 min. Microglia were maintained in DMEM/F12 medium (Gibco, Grand Island, NY, USA) supplemented with 10% fetal bovine serum (FBS) (Gibco) in a humidified incubator at 37°C with 5% CO_2_. BV-2 murine microglial cell lines purchased from the Cell Bank of the Chinese Academy of Sciences (Shanghai, China) were cultured in DMEM/F12 medium with 10% FBS in a humidified atmosphere at 37°C with 5% CO_2_. miR-103a-3p mimics or inhibitors (50 nM), or negative controls (Ruibo Biotechnology, China), were transfected into cells with Lipofectamine 2000 (Invitrogen, Carlsbad, CA, USA). The cells were then treated with LPS (InvivoGen, Hongkong, China; cat no. #tlrl-eblps, final concentration 100 ng/mL diluted in culture medium, 100 *μ*g/mL in-stock (1000x) in distilled water) 1 day after transfection for the indicated time. For dexmedetomidine (Selleckchem, CA, USA, cat no. #S3075) administration, final concentration 1 *μ*M in DMSO was added prior to the addition of LPS.

### 2.2. Animals

Male Sprague-Dawley rats (200–220 g) were purchased from the Laboratory Animal Center of Jinan University (Guangzhou, China). Each group contained three rats. The rats were housed in pathogen-free (SPF) facilities under a 12 h light/dark cycle in temperature- and humidity-controlled rooms. Prior to experimental manipulation, rats were acclimated to the housing facilities for 1 week. All experimental protocols and animal-handling procedures were in accordance with the Guide for the Care and Use of Laboratory Animals from the NIH and the entire animal protocol (including the animal behavior testing) prior to conducting the experiments was approved by the Jinan University Institutional Animal Care and Use Committee (approval number: #2018035). The rats were monitored thrice every day for health status, before or after the surgery or Morris water maze test. When no adverse events were indicated, the animals were weighed again before the initiation of the experiment.

### 2.3. Surgical Trauma Model of POCD

The widely used surgical trauma model of POCD was performed as previously described [27, 28]. Briefly, animals were anesthetized with halothane [29] and then incised longitudinally to a length of 6 cm along the dorsal median line and 5 cm along the abdominal median line. The intestinal tracts were then removed from the abdominal cavity and exposed for 5 min. To prevent infection, Polysporin (Pfizer, Markham, Ontario, Canada) was applied to the surgical area. Sham-operated rats were anesthetized, and the abdominal and dorsal areas were shaved and cleaned. The animals remained under halothane anesthesia for the same amount of time as their surgical counterparts.

### 2.4. Animal Behavior Tests

We applied open-field test (postoperative day 5) and Morris water maze test (postoperative 1-5 days) to detect cognitive changes in the POCD model mice [7, 30]. Briefly, for the Morris water maze test, a platform was placed in the center of a water pool with its top positioned 5 cm above the water surface. Animals were trained for 2 min each time for a total of 6 times, after which the platform was removed and the animals were released at the same entrance as used on postoperative day 1 to day 5. The motion trail, the escape period, and the swimming path were recorded. For the open-field test, rats were placed on an open-top square plywood box (overall dimensions: 30 cm (H), 72 cm length (H), and 72 cm (W)) that had been painted with flat black enamel. Artificial light/dark conditions were used to mimic day and night. During the 5 min the rats spent in the box, the average distance traveled, the traveling path, the bout time, and the time spent in the central region were recorded using a camera mounted above the apparatus. Images were processed using Ethovision XT software (Noldus Information Technology, Wageningen, Netherlands). For dexmedetomidine administration, 50 *μ*g/kg dexmedetomidine dissolved in 2 ml and 0.9% normal saline was injected intraperitoneally.

### 2.5. Dual-Luciferase Reporter Assay

A potential binding site between miR-103a-3p and VAMP1 was predicted using TargetScan [31], miRDB [32], and picTar [33] online software packages. The 3′-UTR sequence of VAMP1 mRNA containing the putative or mutant miR-103a-3p-binding site (VAMP1 3′-UTR-wt and VAMP1 3′-UTR-mut, respectively, approximately 300 nucleotides long) was synthesized by GenePharm (Shanghai, China) and cloned into the pmirGLO vector (Promega, Madison, WI, USA). Cells were co-transfected with the negative control and miR-103a-3p mimic. A dual-luciferase reporter gene assay was conducted after 48 h according to the manufacturer's instructions.

### 2.6. RNA Extraction and Quantitative RT-PCR

Total RNA was extracted with TRIzol (Invitrogen, Carlsbad, CA, USA) as per the manufacturer's instructions and reverse transcribed by the EasyScript First-Strand cDNA Synthesis SuperMix kit (TransGen Biotech, China). A TransScript miRNA First-Strand cDNA Synthesis SuperMix kit (TransGen Biotech, China) was used for miRNA cDNA synthesis. xA TransStart Top Green quantitative PCR (qPCR) SuperMix kit (TransGen Biotech, China) was used to perform quantitative reverse transcription polymerase chain reactions. Relative miR-103a-3p and mRNA expression values were normalized to U6 and glyceraldehyde 3-phosphate dehydrogenase (GAPDH) using the delta-delta-Ct method. Mature miR-103a-3p-specific primers were purchased from Qiagen. The following sense and anti-sense primers were used: miR-103a-3p:5′ − ATCCAGTGCGTGTCGTG − 3′, 5′ − TGCTAGCAGCATTGT ACAGG − 3′; VAMP1: 5′ − ACATGACCAGTAACAGACGACT − 3′, 5′ − ACGTTCA CACGTATGATGTCC − 3′; TNF-*α*: 5′ − ACTGAACTTCGGGGTGATTG − 3′, 5′ − GCT TGGTGGTTTGCTACGAC; IL-1*β*: 5′ − CACCTTCTTTTCCTTCATCTTTG − 3′, 5′ − GTCGTTGCTTGTCTCTCCTTGTA − 3.

### 2.7. Western Blotting

Lysates extracted from BV-2 microglial cells or tissues were resolved on 10% SDS-polyacrylamide gels and transferred to polyvinylidene fluoride (PVDF) membranes [24]. The membranes were blocked with 5% milk and then incubated with recombinant anti-VAMP1 rabbit monoclonal antibody (ab151712, Abcam, Cambridge, UK) diluted 1 : 1000, or anti-GAPDH mouse monoclonal antibody (ab8245, Abcam) diluted 1 : 2000, at 4°C overnight. After washing, the membranes were incubated with secondary antibody (Abclonal Biotechnology, China) for 1 h at room temperature. Proteins were detected by enhanced chemiluminescence (ECL) (Beyotime, China). The original blots could be found in supplemental data.

### 2.8. Enzyme-Linked Immunosorbent Assay (ELISA)

To determine the expression levels of TNF-*α* and IL-1*β* in cell cultures or brain tissues after treatments, supernatants from those samples were tested with an ELISA kit, following the manufacturer's instructions (R&D Biosciences, San Diego, CA, USA).

### 2.9. Statistical Analysis

The data were analyzed using SPSS software 21.0 (SPSS Inc., Chicago, IL, USA). Statistical analysis was performed with analysis of variance (ANOVA) followed by the Bonferroni post hoc test for multiple comparisons. The data are expressed as mean ± standard error from at least three repeated experiments. Unpaired Student's *t*-test was used for single comparisons. A value of *p* < 0.05 was considered statistically significant.

## 3. Results

### 3.1. miR-103a-3p Is Downregulated and Critical for LPS-Induced Microglial Activation

**Figure 1:**
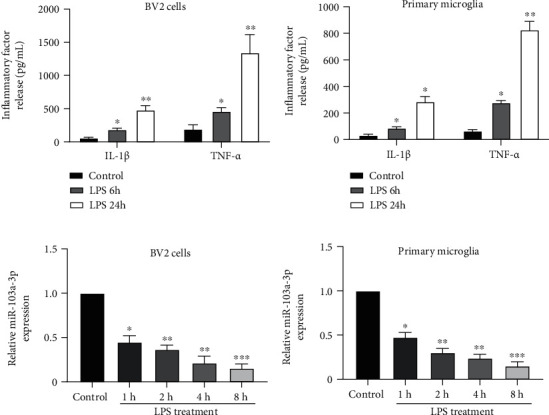
miR-103a-3p is downregulated in LPS-treated BV-2 cells and primary microglial cells. Cultured BV-2 cells (a) and primary microglial cells (b) were treated with 100 ng/ml LPS for 6 h or 24 h (control, distilled water). The supernatants were collected and subjected to ELISA for the detection of IL-1*β* and TNF-*α*. The expression levels of miR-103a-3p were detected by qRT-PCR from cell lysate of BV-2 cells (c) and primary microglia (d). ANOVA, ∗*p* < 0.05, ∗∗*p* < 0.01, ∗∗∗*p* < 0.001, compared with the control group, *n* =4 (number of repeated experiments). IL-1*β*: interleukin-1 beta; LPS: lipopolysaccharide; TNF-*α*: tumor necrosis factor alpha.

**Figure 2:**
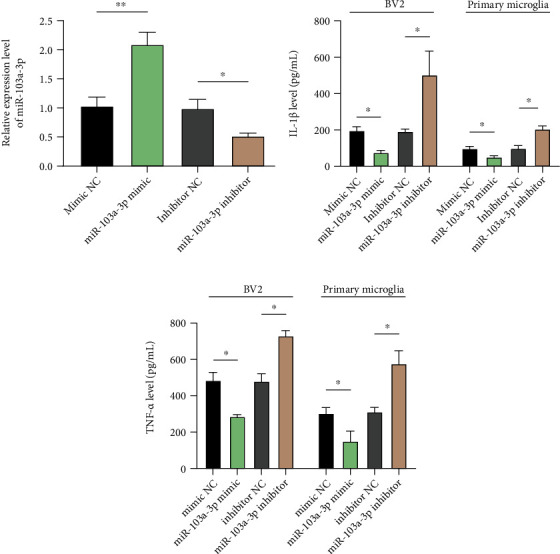
miR-103a-3p is important for microglial activation in BV-2 cells and primary microglia. (a) Synthesized miR-103a-3p mimics and inhibitors were transfected in BV-2 cells, and the miR-103a-3p expression level was then detected by qRT-PCR (with inhibitor NC or mimic NC as controls). Cells were transfected with miR-103a-3p mimics and inhibitors for 12 h, and then treated with LPS for 6 h. IL-1*β* (b) and TNF-*α* (c) levels were then determined with an ELISA (*n* =3, number of repeated experiments). Two tailed *t*-test, ∗*p* < 0.05, ∗∗*p* < 0.01.

Cultured BV-2 cells or primary microglial cells were treated with LPS at dedicated time points, and the supernatants were collected for the detection of inflammatory factors. The levels of IL-1*β* and TNF-*α* were significantly increased in both BV-2 cells and primary microglia, indicating an increased LPS-induced inflammatory response (Figures 1(a) and 1(b)). Moreover, miR-103a-3p expression, measured by qPCR, steadily decreased in response to LPS stimuli in a time-dependent manner (Figures 1(c) and 1(d)). To investigate the role of miR-103a-3p during microglial activation, we synthesized miR-103a-3p mimics and inhibitors. Overexpression of mimics elevated miR-103a-3p levels in BV-2 cells, whereas the inhibitor suppressed miR-103a-3p expression (Figure 2(a)); this verified the efficient function of these fragments. Furthermore, overexpression of miR-103a-3p mimics suppressed, whereas that of inhibitors promoted, IL-1*β* (Figure 2(b)) and TNF-*α* (Figure 2(c)) secretion. These data suggest that miR-103a-3p is critical for the inhibition of microglial activation.

### 3.2. VAMP1 Is a Direct Target of miR-103a-3p

**Figure 3:**
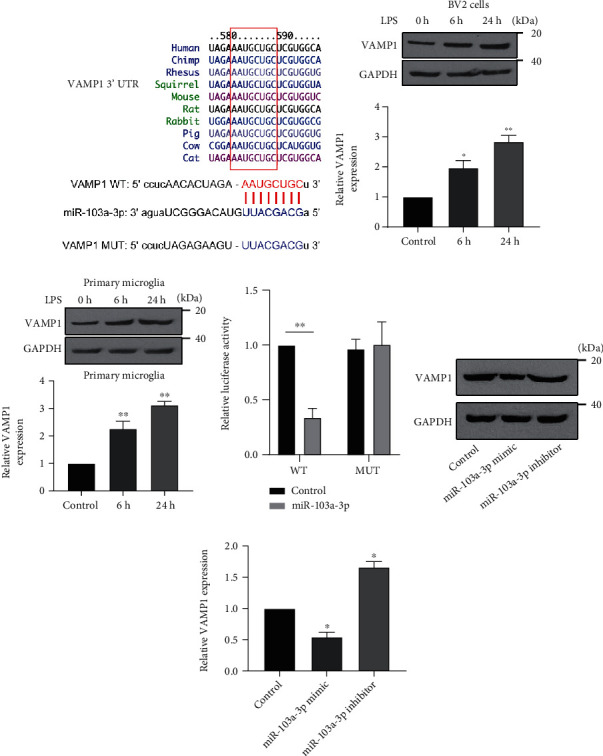
VAMP1 is a direct target of miR-103a-3p. (a) The target site of VAMP1 3′-UTR from different species is shown. VAMP1 WT and MUT luciferase reporter plasmids were constructed. Protein expression levels of VAMP1 after LPS treatment for 6 h and 24 h (0 h as control), and statistical data, are shown for BV-2 cells (b) and primary microglia (c). (d) VAMP1 luciferase reporter plasmids were co-transfected with miR-103a-3p mimic or control (mimic NC sequence). Luciferase activity was measured in cultured BV-2 cells. (e) Protein expression levels of VAMP1 were determined by western blotting of extracts from BV-2 cells transfected with miR-103a-3p mimic or inhibitor (mimic and inhibitor NC as controls) (*n*=3, number of repeated experiments). ANOVA ((b), (c), and (f)) and two tailed *t*-test, ∗*p* < 0.05, ∗∗*p* < 0.01.

To explore the functional downstream target of miR-103a-3p during microglial activation, we first used informatics analysis to screen the database. The results from TargetScan [31], miRDB [32], and picTar [33] online databases showed that VAMP1 was a potential target of miR-103a-3p, and that the matched sequences from the 3′-UTR of VAMP1 were conserved among many vertebrate species (Figure 3(a)). There is, however, no data showing the role of VAMP1 during microglial activation. We first measured the expression level of VAMP1 in LPS-treated cell cultures. VAMP1 levels were high in BV-2 cells and primary microglia (Figures 3(b) and 3(c)), indicating a negative relationship with miR-103a-3p expression. To confirm that miR-103a-3p directly targets VAMP1, we constructed luciferase reporter plasmids encoding the 3′-UTR of VAMP1 WT and MUT (Figure 3(a)). Luciferase activity in BV-2 cells was measured following reporter-transfection with or without miR-103a-3p mimics. WT VAMP1 activity, but not that of the MUT reporter, was significantly suppressed by the miR-103a-3p mimics (Figure 3(d)). Immunoblotting revealed that miR-103a-3p mimics reduced VAMP1 expression while the inhibitors increased it (Figures 3(e) and 3(f)). These data confirm that miR-103a-3p suppresses VAMP1 expression by directly binding to its 3′-UTR.

### 3.3. Dexmedetomidine Alleviates LPS-Induced Microglial Activation via the miR-103a-3p/VAMP1 Axis

**Figure 4:**
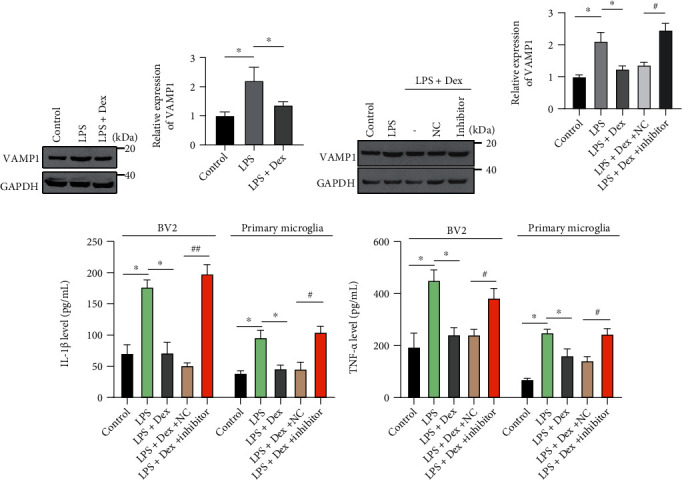
Dexmedetomidine alleviates LPS-induced microglial activation *in vitro*. ((a) and (b)) BV-2 cells were treated with LPS with or without dexmedetomidine (Dex, 1 *μ*M), and cell lysates were then immunoblotted for VAMP1. ((c) and (d)) BV-2 cells were transfected with miR-103a-3p inhibitor or control, and treated with LPS with or without dexmedetomidine; the VAMP1 expression level was then determined. BV-2 cells or primary microglial cells were treated as in ((c) and (d)), and expression levels of IL-1*β* (e) and TNF-*α* (f) were then determined by ELISA (DMSO as control, *n* =3, number of repeated experiments). Two tailed *t*-test, ∗, #*p* < 0.05, ∗∗, ##*p* < 0.01.

Earlier work revealed a role for dexmedetomidine in LPS-induced neuronal dysfunction [24]; however, the mechanism underlying microglia activation requires further elucidation. In LPS-exposed BV-2 cells treated with dexmedetomidine, the *α*2 adrenoceptor agonist significantly suppressed LPS-induced VAMP1-upregulation (Figures 4(a) and 4(b)). Cells were then transfected with either miR-103a-3p inhibitors or the control prior to LPS and dexmedetomidine treatment. Dexmedetomidine mitigated LPS-induced upregulation of VAMP1, and these were blocked by the overexpression of miR-103a-3p inhibitors (Figures 4(c) and 4(d)). Furthermore, dexmedetomidine treatment significantly decreased the secretion of inflammatory factors IL-1*β* (Figure 4(e)) and TNF-*α* (Figure 4(f)), induced by LPS in both BV-2 and primary microglial cells; this was reversed by prior transfection with miR-103a-3p inhibitors (Figures 4(e) and 4(f)). Dexmedetomidine, therefore, may alleviate LPS-induced microglia activation by modulating the miR-103a-3p/VAMP1 axis.

### 3.4. Dexmedetomidine Regulated miR-103a-3p/VAMP1 Mediates Microglial Activation in a POCD Rat Surgical Trauma Model

**Figure 5:**
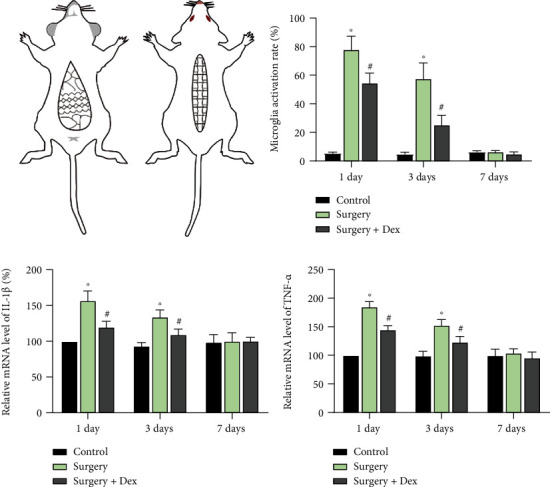
The establishment of a rat surgical trauma model of POCD. (a) Simplified diagram of the rat surgical trauma model. Rats were cut open longitudinally to a length of 6 cm along the dorsal median line and 5 cm along the abdominal median line; the intestinal tracts were exposed for 5 min. (b) The microglial activation rate was determined from Iba1-stained microglia in the rat hippocampus for the times indicated, with or without dexmedetomidine. The mRNA expression levels of IL-1*β* (c) and TNF-*α* (d) were determined (Sham as control, 3 animals for each group, *n* =3 repeated experiments). ANOVA and two tailed *t*-test, ∗*p* < 0.05, compared with control group; #*p* < 0.05, compared with surgery group.

**Figure 6:**
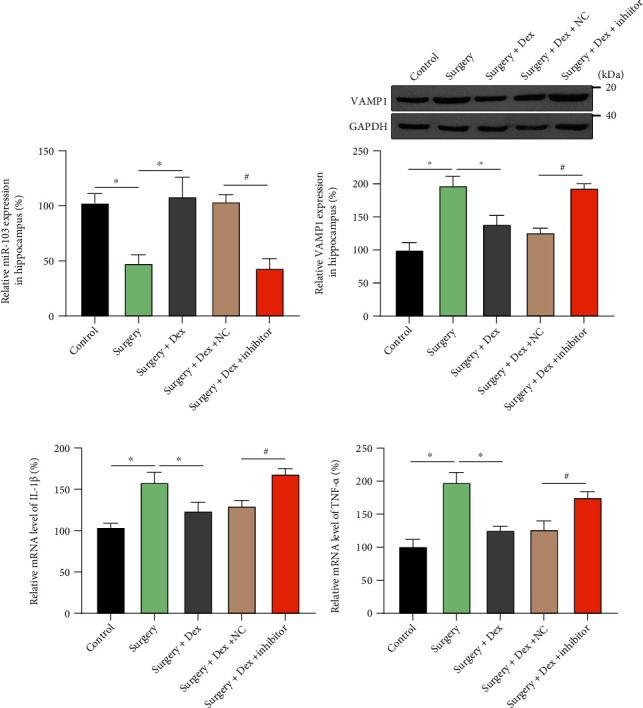
Dexmedetomidine alleviates surgical trauma-induced microglial activation *in vivo*. Rats were treated with or without dexmedetomidine (Dex) and an exogenous miR-103a-3p inhibitor. (a) miR-103a-3p expression was detected by qRT-PCR. (b) VAMP1 protein expression in the hippocampus was detected by immunoblotting; mRNA expression levels of IL-1*β* (c) and TNF-*α* (d) were determined in the hippocampus (Sham as control, 3 animals for each group, *n* =3 repeated experiments). Two tailed *t*-test, ∗, #*p* < 0.05.

**Figure 7:**
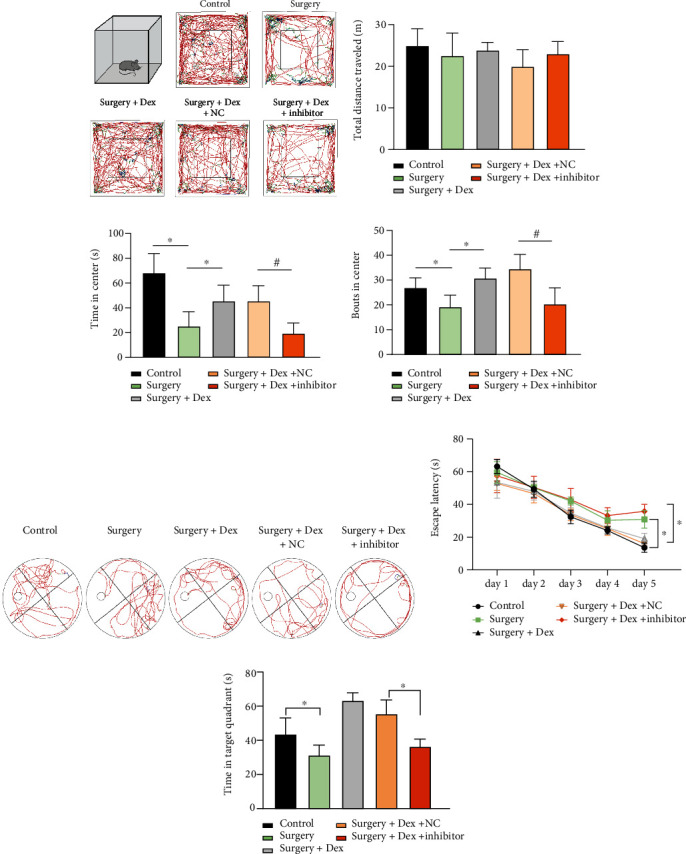
Changes in rat behavior following surgical trauma. ((a)–(d)) Open-field tests. Rats were treated as described in Figure 6 and then subjected to open-field tests. (a) Typical animal routes are shown. The total distance traveled (b), the time spent in the center (c), and bout time in the center (d) were analyzed. ((e)–(g)) Morris water maze tests. Postoperative rats were subjected to water maze tests for five days. The swimming path was recorded and typical images are shown (e). Escape latency was calculated each day (f) and the time spent in the target quadrant was calculated for each group (g). (Sham as control, 3 animals for each group, *n* =3 repeated experiments). ANOVA (f) and two tailed *t*-test, ∗, #*p* < 0.05.

To investigate the roles of dexmedetomidine and miR-103a-3p/VAMP1 during microglial activation *in vivo*, a rat surgical trauma model of POCD was established as previously reported (Figure 5(a)) [27, 28]. The surgical procedure produced significant microglial activation in the hippocampus, a region associated with cognitive dysfunction during POCD. Dexmedetomidine treatment significantly reduced microglial activation (Figure 5(b)). We further detected the mRNA expression of genes linked to microglial activation and inflammation in the hippocampus. IL-1*β* (Figure 5(c)) and TNF-*α* (Figure 5(d)) expressions were significantly increased post-surgery, but were downregulated in response to dexmedetomidine administration. In the hippocampus, and similar to the response to LPS treatment *in vitro*, miR-103a-3p expression decreased (Figure 6(a)) and that of the downstream target VAMP1 increased (Figure 6(b)). Dexmedetomidine treatment significantly reduced miR-103a-3p downregulation and VAMP1 upregulation; these effects, however, were not observed following pretreatment with miR-103a-3p inhibitors (Figures 6(a) and 6(b)). IL-1*β* (Figure 6(c)) and TNF-*α* (Figure 6(d)) mRNA levels showed the same trend as VAMP1. Furthermore, we used open-field tests to examine the effect of dexmedetomidine treatment on animal behavior, because anxiety and depression are also cognitive symptoms of POCD. [34, 35] The data of total distance showed that dexmedetomidine administration did not reduce animal activity; however, surgery significantly reduced the time spent in the center and the bout time in the center (Figure 7(a)). Dexmedetomidine abolished this phenomenon, and this effect of dexmedetomidine was reversed by miR-103a-3p inhibitor treatment (Figures 7(b) and 7(c)). Furthermore, we used the Morris water maze test to detect changes in memory and learning after surgery. As shown in Figures 7(e)–7(g), surgery affected learning efficiency and resulted in a longer escape latency (f) and a shorter time in the target quadrant (g). Dexmedetomidine mitigated these effects of surgery, and this effect of dexmedetomidine was reversed by miR-103a-3p inhibitor treatment (Figures 7(f) and 7(g)). Taken together, these results suggest that miR-103a-3p/VAMP1 mediates the protective effect of dexmedetomidine against surgery-induced microglial activation *in vivo* and that targeting of miR-103a-3p/VAMP1 suppresses microglial activation-related inflammation in POCD.

## 4. Discussion

The present study shows that miR-103-3p and its downstream target VAMP1 play an important role in microglial activation, both *in vitro* during LPS treatment and surgical trauma-induced POCD *in vivo*. Furthermore, we show that dexmedetomidine increased miR-103a-3p levels and ameliorated microglial activation *in vivo* and *in vitro*, thereby preventing VAMP1-mediated proinflammatory cytokine release. Targeting the miR-103a-3p/VAMP1 axis may reduce microglial activation-induced neuroinflammation and provide a new strategy for the clinical treatment of POCD.

Microglial activation induced the release of inflammatory factors, causing neuronal apoptosis and impairing synaptic plasticity. Microglial activation is a major factor in the development of post-surgery psychiatric disorders, particularly POCD. It is, therefore, crucial to understand the underlying mechanism of microglial activation in POCD. miRNAs including miR-124, miR-146a, and miR-181b-5p are critical for regulating microglial development and activation. [36–38] miR-103a-3p is linked with cancer progression [39–41] but also performs a variety of physiological functions across multiple biological processes, including immunity [42] and oxidative stress. [43] miR-103a-3p is significantly downregulated in LPS-treated lung cells; miR-103a-3p overexpression reduces LPS-induced inflammation, [42, 44] showing a protective effect of these non-coding RNA molecules against inflammation. [43] The contribution of miR-103a-3p to neurological diseases, however, has rarely been reported. miR-103a-3p, in particular, is involved in the gene regulatory network associated with autism spectrum disorder in China, [45] and expression of miR-103a in the blood is found in L-dopa-treated patients with Parkinson's disease. [46] Furthermore, miR-103 promotes neurite outgrowth and inhibits apoptosis in a cell culture model of AD, [47] and alleviates autophagy and apoptosis in LPS-injured PC12 cells in a rat model of spinal cord injury. [48] As miR-103 is an important anti-inflammatory and neuroprotective molecule, we hypothesized that miR-103a-3p would be involved in CNS inflammation. In the present study, we investigated miR-103a-3p expression levels in LPS-treated BV-2 cells and in surgical trauma-induced POCD rats; miR-103a-3p was significantly downregulated during microglial activation. miR-103a-3p gain-and-loss functional assays revealed that the protective function of miR-103a-3p is the inhibition of inflammatory cytokine release via modulation of its direct target VAMP1.

VAMP1, a member of the synaptobrevin family, is involved in synaptic vesicle docking and fusion. [49] VAMP1 is also a core component of the SNARE (soluble N-ethylmaleimide-sensitive factor attachment protein receptors) complex that mediates intracellular vesicular trafficking. [50] VAMP1 acts as a v-SNARE and interacts with SNAP-25 and syntaxin1 to form a stable complex for vesicular fusion and neurotransmitter release. [51] VAMP1 has also been linked to a number of neurological disorders. Increased VAMP1 expression is linked with a high risk of AD, whereas lower expression has a protective effect. [52] VAMP1 may therefore be associated with cognitive function. In the current study, VAMP1 and inflammatory cytokine expression were increased in LPS-treated cells in culture and in the rat surgical trauma model. The role of VAMP1 during neuronal inflammation and cognitive dysfunction, particularly in POCD, however, requires further investigation.

Surgical trauma induces an inflammatory response through innate immunity. [53] Following trauma, damage-associated molecular patterns (DAMPs), including TNF-*α*, IL-1*β*, and other factors, are upregulated and mediate the inflammatory response via signaling through a variety of evolutionarily conserved pattern recognition receptors (PRRs); [54] this further increases the synthesis and release of proinflammatory mediators, [55] leading to neuroinflammation and cognitive decline. [56] In the present study, surgical trauma decreased miR-103a-3p expression in the hippocampus from rats with POCD, and concomitantly aggravated microglial activation together with upregulation of inflammatory cytokines such as IL-1*β* and TNF-*α*; this indicates the potential role of miR-103a-3p in POCD. However, further research involving the overexpression or inhibition of miR-103a-3p in surgical trauma-exposed animal models is needed to confirm its critical role in POCD.

Dexmedetomidine, an *α*2-agonist, is frequently used during surgical procedures [57] and has anti-inflammatory properties; [58] however, the underlying mechanisms are still under investigation. Dexmedetomidine increases miR-340 expression and regulates glycolysis in LPS-stimulated BV-2 cells [59, 60] and reduces neuroinflammation by modulating programmed cell death protein 1. [61] Dexmedetomidine administration improves cognitive dysfunction in aged mice with early-stage POCD. [62, 63] Moreover, this compound facilitates neuronal NOS (nNOS) expression in hippocampal neurons and alleviates surgical trauma-induced neuroinflammation and cognitive dysfunction in aged rats. [64] Dexmedetomidine treatment, furthermore, protects hippocampal neuron from apoptosis and attenuates cognitive impairment in transgenic mouse models of AD, via the miR-129/YAP1/JAG1 axis. [65] Dexmedetomidine ameliorates POCD via the miR-381/EGFR1/p53 axis [66] and is protective against sevoflurane-induced postoperative cognitive dysfunction through the miR-129/TLR4 axis. [67] In the current study, dexmedetomidine treatment reduced microglial activation in LPS-stimulated BV-2 cells and primary microglia cells, and in a rat model of surgical trauma that mimicked POCD. Dexmedetomidine increased miR-103a-3p expression but decreased VAMP1 levels. The proactive effect of dexmedetomidine was eliminated by modulating the levels of miR-103a-3p. However, direct evidence of cognitive function in rats, mediated through the miR-103a-3p/VAMP1 axis and dexmedetomidine, must be supplemented with additional studies.

In summary, our findings demonstrate the involvement of miR-103a-3p and VAMP1 during microglial activation and subsequent inflammatory responses. The data further show that dexmedetomidine protects against POCD. *In vitro* and *in vivo*, dexmedetomidine alleviates inflammation via the miR-103a-3p/VAMP1 axis. The miR-103a-3p/VAMP1 axis may be a novel therapeutic target for intervention against surgery-related microglial activation. In addition, dexmedetomidine has an epigenetic effect on neuroprotection in POCD, which provides new insights for the development of novel therapies for the prevention of POCD.

## 5. Conclusions

In general, the present data show that miR-103a-3p is critical for microglial activation *in vitr*o and *in vivo*. VAMP1 is a direct target of miR-103a-3p. In a surgical trauma model of POCD, miR-103a-3p/VAMP1 underpins microglial activation; this suggests a potential therapeutic target for post-surgical disorders associated with microglial activation.

## Data Availability

The data used and/or analyzed during the current study are available from the corresponding author upon reasonable request.

## Abbreviations

AD: Alzheimer's disease
CNS: Central nervous system
mEPSCs: Miniature excitatory postsynaptic currents
PD: Parkinson's disease
POCD: Postoperative cognitive dysfunction.
